# Multimodal MRI Neuroimaging Biomarkers for Cognitive Normal Adults, Amnestic Mild Cognitive Impairment, and Alzheimer's Disease

**DOI:** 10.1155/2012/907409

**Published:** 2011-09-21

**Authors:** Ai-Ling Lin, Angela R. Laird, Peter T. Fox, Jia-Hong Gao

**Affiliations:** ^1^Research Imaging Institute, The University of Texas Health Science Center at San Antonio, 7703 Floyd Curl Drive, San Antonio, TX 78229, USA; ^2^Departments of Radiology, Psychiatry, and Behavioral Neuroscience and Brain Research Imaging Center, The University of Chicago, 5841 South Maryland, MC 2026, Chicago, IL 60637, USA

## Abstract

Multimodal magnetic resonance imaging (MRI) techniques have been developed to noninvasively measure structural, metabolic, hemodynamic and functional changes of the brain. These advantages have made MRI an important tool to investigate neurodegenerative disorders, including diagnosis, disease progression monitoring, and treatment efficacy evaluation. This paper discusses recent findings of the multimodal MRI in the context of surrogate biomarkers for identifying the risk for AD in normal cognitive (NC) adults, brain anatomical and functional alterations in amnestic mild cognitive impairment (aMCI), and Alzheimer's disease (AD) patients. Further developments of these techniques and the establishment of promising neuroimaging biomarkers will enhance our ability to diagnose aMCI and AD in their early stages and improve the assessment of therapeutic efficacy in these diseases in future clinical trials.

## 1. Introduction

Aging is the greatest risk factor for neurodegenerative disorders in general, but specifically for Alzheimer's disease (AD). With the increasing life expectancy in developed countries, the incidence of AD, and consequently its socioeconomic impact, is growing. AD currently affects about 4.5 million Americans, which costs the USA economy more than $100 billion each year. The number of AD patients is projected to increase to 11–16 millions by 2050, with a cost exceeding $380 billion per year [1, 2]. 

To identify AD and monitor disease progression, neuropsychological tests such as the Mini-Mental State Exam (MMSE) and the cognitive subscale of the Alzheimers disease assessment (ADAS Cog) [3] are currently the most commonly used strategies. However, these tests have several limitations, as follows. MMSE is criticized by its marginal or absent assessment of some cognitive abilities that are affected early in the course of Alzheimer's disease or other dementing disorders (e.g., limited memory and verbal fluency items and no problem solving or judgment items), and its relative insensitivity to very mild cognitive decline, particularly in highly educated individuals. ADAS Cog is limited by its relatively poor test-retest reliability, which likely reflects the influence of other factors on the patients' performance (e.g., the patients' mood). Furthermore, these tests are not able to distinguish the risk for AD in preclinical groups (cognitively normal elderly adults) or predict the conversion to AD from preclinical and mild cognitive impairment (MCI) groups.

The National Institute of Aging (NIA) has recently announced the revised clinical diagnostic criteria for AD dementia for the first time in 27 years (http://www.nih.gov/news/health/apr2011/nia-19.htm). Instead of addressing the disease and describing only later stages when symptoms of dementia are already evident, the updated guidelines cover the full spectrum of the disease as it gradually changes over many years. They describe (i) the earliest preclinical stages of the disease, (ii) MCI, and (iii) dementia due to Alzheimer's pathology. Importantly, the guidelines now address the use of imaging and biomarkers in blood and spinal fluid that may help determine whether changes in the brain and those in body fluids are due to AD. 

In this paper, we will focus on the imaging biomarkers as addressed in the new criteria. Specifically, we will discuss the surrogate biomarkers developed by the multimodal magnetic resonance imaging (MRI) methods for identifying the risk for AD in normal cognitive (NC) adults; brain anatomical and functional alterations in amnestic MCI (aMCI) and AD patients.

## 2. Brief Overview of Multimodal MRI Neuroimaging Biomarkers

### 2.1. Structural Biomarkers

The high spatial resolution, sensitivity, and specificity of MRI (e.g., resolution: 0.8 mm isotropic; sensitivity: 80–94%; specificity: 60–100%) have made it a powerful tool to identify structural alterations and brain atrophy using volumetric measurements of the entire brain [4, 5]. With advanced computer software, the neocortex of the brain on the MRI scans can be automatically subdivided into 32 gyral-based region of interests (ROIs) per hemisphere, including gray matter (GM), white matter (WM), and hippocampus volumes [6–8]. 

**Figure 1:**
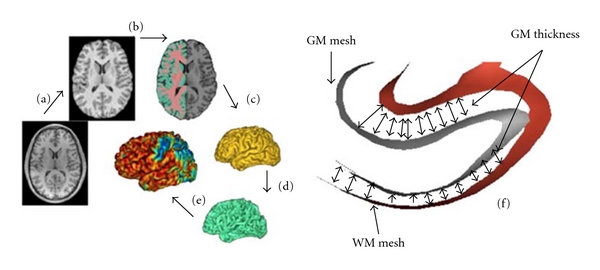
T_1_-weighted image processing pipeline consists of (a) skull stripping; (b) spatial normalization, RF homogeneity correction, and tissue segmentation; (c, d) extraction of GM and WM pial surfaces; (e, f) calculation of GMT.

GM loss can also be determined by measuring cortical gray matter thickness (GMT). GMT is determined by calculating the three-dimensional distance from the outer cortical surface to the inner cortical GM-WM boundary using cortical modeling from the high-resolution MRI structural images (Figure 1). WM integrity can be assessed with diffusion tensor imaging (DTI). In brain tissues, the microscopic motion of water molecules is hindered by boundaries of tissue structure. In highly structured tissue such as WM, this motion is highly anisotropic and DTI provides directional information about it. Loss in WM structure results in the loss in anisotropy, which can be easily detected by DTI [9, 10].

### 2.2. Functional Biomarkers

Functional-based MRI can detect alterations and monitor disease progression related to brain metabolism, hemodynamics, and connectivity. Functional connectivity MRI (fcMRI) has been developed as a technique to determine the resting state brain connectivity as measured by the basal blood oxygenation-level-dependent (BOLD) signal. In its simplest form, functionally connected networks can be identified using a seed-based correlational approach, in which the average resting state time series from a region of interest is correlated with all other voxels in the brain [2, 11–14]. In contrast to this correlational method, independent component analysis (ICA) is a more advanced multivariate analysis method that allows resting state fMRI data to be decomposed into sets of independent, intrinsic brain networks [15–17]. In either approach, each functional network's neuronal activity is associated with a hemodynamic response, which consists of an increase in cerebral blood flow (CBF) and oxyhemoglobin and a relative decrease in deoxyhemoglobin. The changes in the oxyhemoglobin-deoxyhemoglobin ratio result in changes in BOLD signal. 

Neuronal activity is tightly coupled with CBF (as mentioned above); therefore, another approach to assess the disease progression of AD is to measure CBF (in the units of mL/100 g/min). MR-based CBF measurements have been developed to investigate hemodynamic alteration in AD, including arterial spin labeling (ASL) [18] and dynamic contrast techniques [19]. Compared with the traditional CBF measurements using single-photon emission computed tomography (SPECT) with Tc-99m radioactive tracers [20, 21], the absence of ionizing radiation or injection and the ability to obtain high quality anatomical images within the same scanning session make MRI-based CBF techniques attractive methods for the study of AD, especially when repeated scans are needed for monitoring disease progression or assessing treatment effect. 

Changes in neuronal activity during the progression of AD may be associated with the changes in brain metabolism. Brain metabolism can be measured with MR spectroscopy (MRS). Using proton (^1^H) MRS, numerous metabolites related to brain functions can be determined, including N-acetyl aspartate (NAA), myoinositol (MI), creatine, choline, glutamate, glutamine and lactate. NAA is present only within neural cell body, axons and dendrites, it is thus considered to be a marker of neuronal viability and function [22]. MI, on the other hand, has considerably higher concentration in glial cells and thus is often taken as a glial marker [23].

## 3. Preclinical-Cognitively Normal Adults

Beyond age, family history is the most significant risk factor for AD, with maternal transmission being significantly more frequent than paternal transmission [24]. Biomarkers for AD-associated pathological changes, including metabolic deficits and amyloid beta (A*β*) load, have been observed in cognitively normal individuals who have maternal history of late-onset AD (FHm) [25, 26].

**Figure 2:**
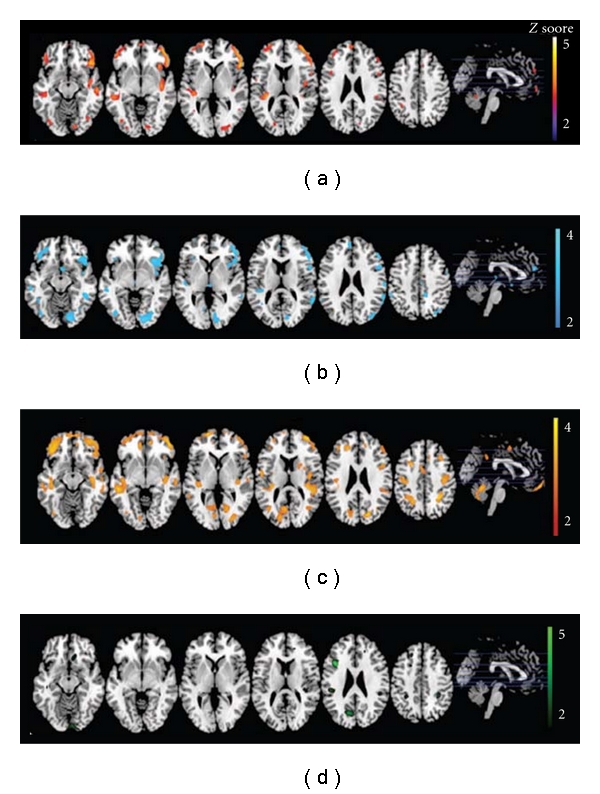
The first 2 rows display maps from subjects with a maternal family history of Alzheimer's disease (FHm) as compared with subjects with no family history (FH-) (a) and subjects with a paternal family history (FHp) (b). Row (c) shows gray matter volume (GMV) reductions in APOE *ε*4-negative FHm subjects compared with APOE *ε*4-negative FH subjects. Statistical parametric maps showing GMV reductions in normal FHp subjects as compared with FH subjects are in row (d). Areas of GMV decrease are represented on purple-to-yellow, blue-to-light blue, dark orange-to-yellow, and green-to-light green color-coded scales for the 4 contrasts, reflecting *Z* scores between 2 and 5 for the upper contrast and between 2 and 4 for the lower 3 contrasts. Areas of gray matter volume decrease are displayed on a standardized spatially normalized MRI (adapted with permission from [27]).

**Figure 3:**
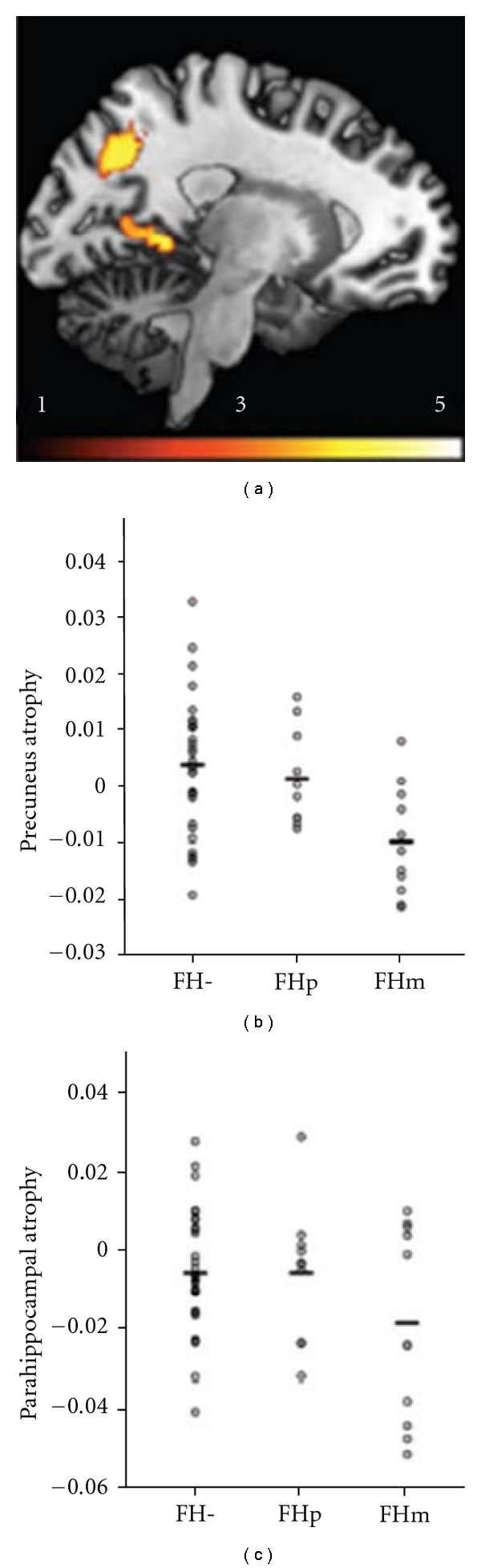
Increased regional atrophy in maternal history of Alzheimer's disease (FHm) group compared to subjects without family history of late-onset Alzheimer's disease (FH-) and paternal history of Alzheimer's disease (FHp) groups (adapted with permission from [28]).

Structural MRI has been used to assess brain volume changes for cognitively intact elderly individuals with FHm, a paternal history of AD (FHp) and no parental history of AD (FH-) [27–29]. Compared with FH individuals, cognitively healthy subjects with a family history of late-onset AD had significantly decreased gray matter volume (GMV) in the precuneus, middle frontal, and superior frontal gyri (Figure 2; [27]). FHm subjects had even significantly smaller inferior frontal, middle frontal, precuneus, and lingual gyri compared with FH and FHp individuals (Figures 2 and 3) [27, 28].

**Figure 4:**
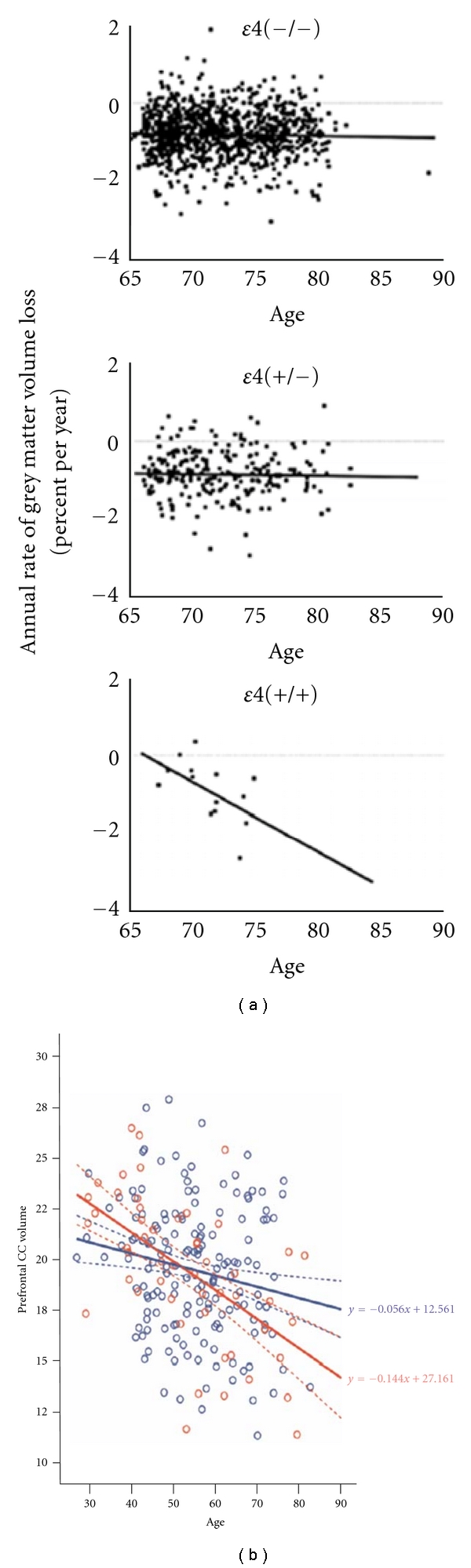
(a) Age effect on the longitudinal followup of GMV for the three APOE *ɛ*4 groups, illustrating the significant interaction between age and the APOE *ɛ*4. *ɛ*4 (–/–): noncarriers for the APOE *ɛ*4 allele, and *ɛ*4 (+/–): heterozygous for the APOE *ɛ*4 allele, *ɛ*4 (+/+): homozygous for the APOE *ɛ*4 allele (adapted with permission from [30]). (b) Regression of the normalized volume of the prefrontal callosal subregion on age in APOE *ε*4 carriers (red) and noncarriers (blue). Thick lines show linear regression lines and dotted lines 95% mean confidence intervals of the slope. Formulas denote regression lines. Normalization was done to total intracranial volume (adapted with permission from [31]).

Another chief known genetic factor for AD is *ε*4 allele apolipoprotein E gene (APOE *ε*4). Increasing age and carrying APOE *ε*4 are well-established risk factors for AD. Healthy older APOE *ε*4 carriers, particularly *ε*4 homozygotes, have demonstrated brain structure changes related to noncarriers. In a recent study with a longitudinal cohort of 1186 healthy elderly persons (65–89 years), Crivello et al. found that an annual rate of gray matter volume loss was seen in *ε*4 homozygotes, whereas no age effect was seen in *ε*4 heterozygotes and in noncarriers (Figure 4(a)) [30]. Similarly, *ε*4 homozygotes had a significant larger rate of hippocampal volume loss than heterozygotes or noncarriers. In another anatomical study, Filippini et al. observed white matter atrophy, including corpus callosum (CC) volume and all subregions, in both APOE *ε*4 carriers and noncarriers. However, the slope has been steeper in the APOE *ε*4 carriers compared with the noncarriers particularly in the prefrontal region (*P* = 0.02) (Figure 4(b)) [31].

**Figure 5:**
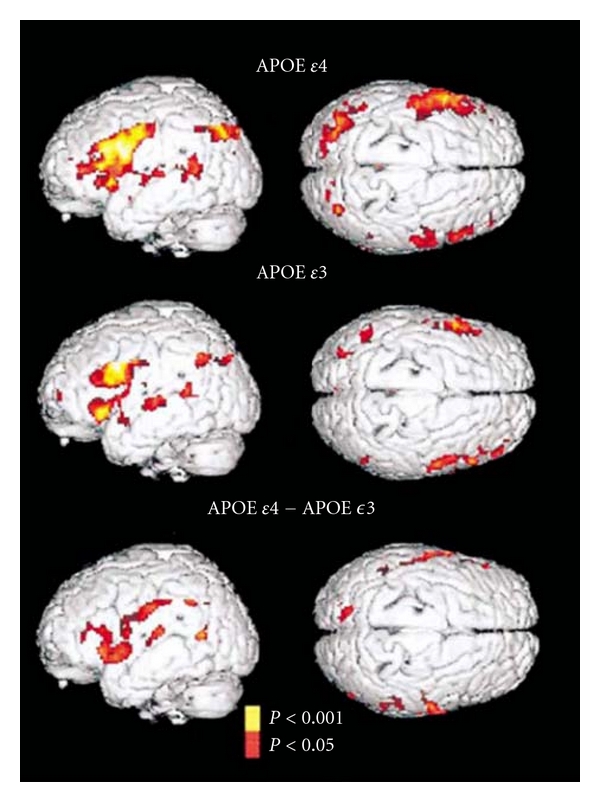
Statistical parametric maps of the brain used to assess subjects' performance on memory-activation tests in carriers of the APOE *ε*4 allele and carriers of the APOE *ε*3 allele. The signal intensity increased significantly in the left inferior frontal region, the right prefrontal cortex, the transverse temporal gyri bilaterally, and the left posterior temporal and inferior parietal regions in both groups. However, both the extent and the intensity of activation were greater among the carriers of the APOE *ε*4 allele. The carriers of the APOE *ε*4 allele also had significant increases in the left parahippocampal, the left dorsal prefrontal cortex, and in the inferior and superior parietal lobes and the anterior cingulate gyrus. Direct comparisons of the carriers of the APOE *ε*4 allele and the carriers of the APOE *ε*3 allele (bottom panel, which shows the difference between the carriers) further demonstrated the greater extent and magnitude of activity in the left prefrontal region and bilateral orbitofrontal, superior temporal, and inferior and superior parietal regions in the carriers of the APOE *ε*4 allele (adapted with permission from [32]).

In addition to structural changes, APOE *ε*4 has also shown great impact on brain function. Memory is the first cognitive domain to be affected by AD [42], and impairments have been found in APOE *ε*4 carriers relative to noncarriers [43, 44]. Using functional MRI (fMRI), Bookheimer et al. observed that during a memory task in a group of healthy subjects (aged 47–82), APOE *ε*4 carriers demonstrated significant increases in the left parahippocampal region, the left dorsal prefrontal cortex, the inferior-superior parietal lobes, and the anterior cingulate gyrus (Figure 5; [32]). In addition, the extent and the intensity of activation for the APOE *ε*4 carriers were greater in the left inferior frontal region, the right prefrontal cortex, the transverse temporal gyri bilaterally, the left posterior temporal, and inferior parietal regions relative to the noncarriers (carriers of the APOE *ε*3 allele). Direct comparisons of APOE *ε*4 carriers and noncarriers further demonstrated the greater extent and magnitude of activity in the left prefrontal, bilateral orbitofrontal, and superior temporal regions. In carriers of the APOE *ε*4 allele, it has been demonstrated in inferior and superior parietal regions.

**Figure 6:**
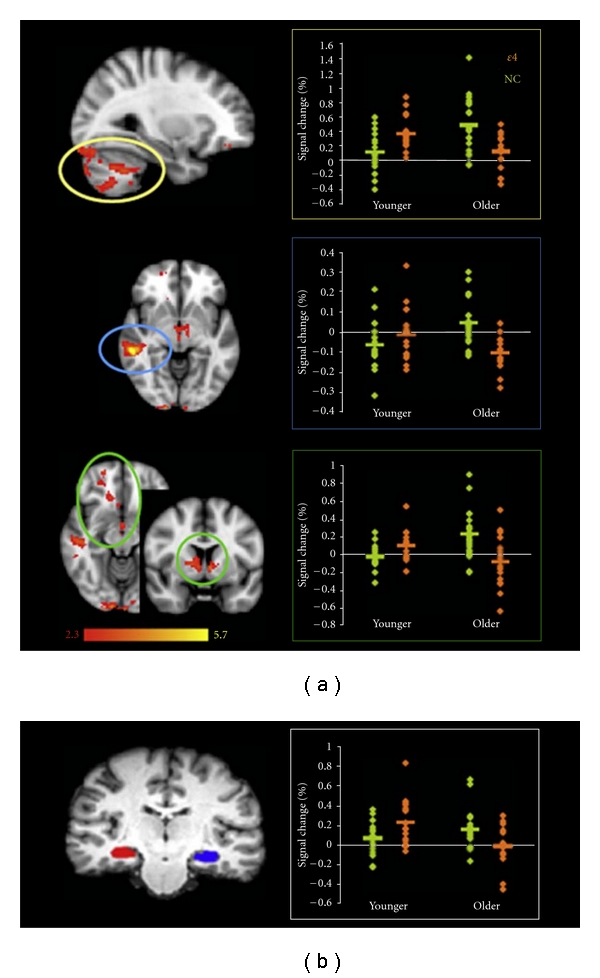
AGE by GENE interactions in the “novel versus familiar” contrast of the encoding task. (a) Regions showing significant interaction between AGE and GENE factors (*P* < 0.05, corrected for multiple comparisons) with plots of percentage signal change in brain regions showing group-related differences where *ε*4 (orange) defines *ε*4-carriers and NC (green) defines noncarriers. (b) ROIs for the left and right hippocampi overlaid on a structural image (left) with associated plot of average hippocampal percentage signal change showing significant age-by-gene interaction (left hippocampus: *P* = 0.002, right hippocampus: *P* = 0.003) (adapted with permission from [33]).

In a younger group (mean age = 21–30), APOE *ε*4 carriers demonstrated increased task-induced brain activation in hippocampus relative to the noncarriers [45, 46]. Overactivity of brain function has also been found in young APOE *ε*4 carriers but disproportionately reduced with advancing age even before the onset of measurable memory impairment (Figure 6; [33]). In both age groups, a significant interaction has been found between age and APOE *ε*4 status in the hippocampi, frontal pole, subcortical nuclei, middle temporal gyri, and cerebellum. These results have suggested that APOE genotype determines age-related changes in brain function, and greater activation reflects greater cognitive “effort” by APOE *ε*4 carriers to obtain the same level of performance as the noncarriers, and/or reflect neuronal mechanism to compensate for processes, such as reduced synaptic plasticity, neuronal growth, or altered long-term potentiation in the carriers. 

APOE *ε*4 carriers have also shown disrupted resting state brain activity in the absence of A*β* or decreased CSF in cognitively normal elderly (mean age = 62) using functional connectivity MRI method [11–13, 47]. Similarly, young APOE *ε*4 carriers (mean age = 21–30), although had no difference in cognition and GM volume compared to their age-mated controls, showed increase in default mode network (involving medial temporal, medial prefrontal, and retrosplenial cortical areas) coactivation [46], suggesting that the function of these areas subject to the disease process in AD is modulated by APOE *ε*4 allele at very early stage. 

Taken together, these results provide evidence that influence of the genetic effect (familial and APOE *ε*4 allele) on neurophysiological characteristics and the risk for AD can be detected using MRI decades prior to any clinical or neuropathological expression of neurodegenerative process.

## 4. Mild Cognitive Impairment

MCI is a transitional state between normal aging and dementia. MCI is a diagnosis given to individuals who experience memory problems greater than normally expected with typical aging, but who do not show other symptoms of dementia, such as impaired judgment or reasoning. MCI has various clinical subtypes, including amnestic single domain (aMCI-S), amnestic multiple domain (aMCI-M), nonamnestic single domain (naMCI-S), and nonamnestic multiple domain (naMCI-M) [48]. Nonamnestic forms of MCI (naMCI, i.e., naMCI-S and naMCI-M) have had findings suggestive of vascular disease, whereas amnestic forms of MCI (aMCI, i.e., aMCI-S and aMCI-M) have appeared to have demographic, genetic, and MRI findings suggestive of AD pathology [48, 49]. Although aMCI can be defined using neuropsychiatric criteria, brain imaging studies have aimed to develop measures that are sensitive enough to distinguish aMCI from normal aging with high specificity [50]. Many other studies attempt to differentiate between aMCI subjects who will convert to AD, over a specific followup interval versus those who remain stable or ever recover [51]. In this section, we elaborate the current findings in these two areas.

### 4.1. Distinguishing aMCI from Normal Aging

**Figure 7:**
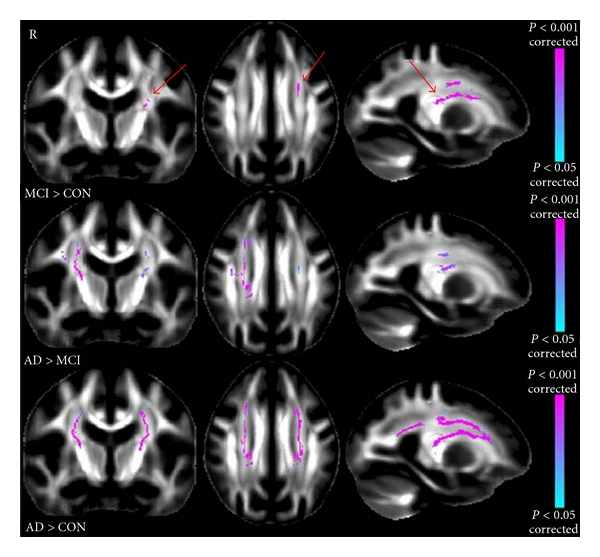
*Top.* Significant mode of atrophy (MO) results showing the contrast MCI > CON (controls; arrows). *Middle.* Significant MO results showing the contrast AD > MCI. *Bottom.* Significant MO results showing the contrast AD > CON. (adapted with permission from [34]).

**Figure 8:**
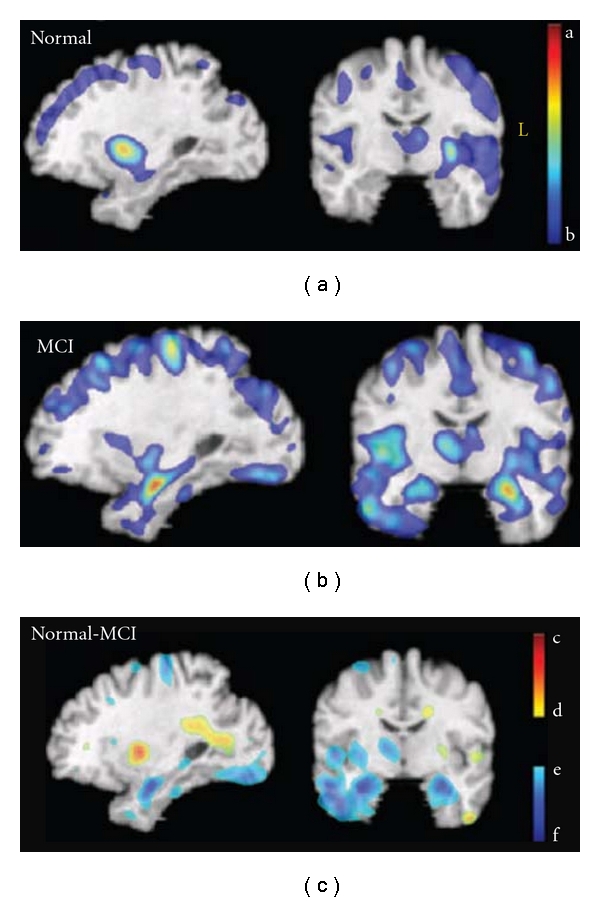
Patterns of GMV loss in MCI and normal aging. Average slopes of RAVENS maps for normal (a) and MCI (b) groups. The red-yellow color indicates greater volume loss. Bottom row: difference between the two groups; blue/green are regions in which MCI subjects showed higher rate of gray matter decrease. (c) Red/yellow colors reflect an increase of periventricular small vessel disease, which appears gray in T1-weighted images and is segmented as gray matter. The color bars display estimated regression coefficients and are defined by the following numbers, all in mm^3^/year (per voxel in the template space): a = −0.020, b = −0.0053, c = 0.026, d = 0.0053, e = −0.0053, and f = −0.023 (adapted with permission from [35]).

Although age-related regional volume loss is apparent and widespread in nondemented individuals [5, 52], aMCI is associated with a unique pattern of structural vulnerability reflected in differential volume loss in specific regions. In a cross-sectional study, aMCI patients were observed with a significant WM abnormality in the region of crossing fibers in the centrum semiovale in comparison to NC (Figure 7) [34]. In a ten-consecutive-year longitudinal study, 18 participants (among 138) who converted from normal to MCI showed accelerated changes (compared to normal controls) on whole brain volume, ventricular CSF (vCSF), temporal gray matter, and orbitofrontal and temporal association cortices, including the hippocampus (*P* ≤ 0.04) (Figure 8) [35]. 

Similar findings of vCSF increases in aMCI patients compared to normal controls have been reported by Vemuri et al. [53]. In this study, Vemuri and colleagues further demonstrated that changes in serial structural MRI differed by APOE *ε*4 status overall among aMCI, with higher brain atrophy rates in APOE *ε*4 carriers. In addition, MR-based structural biomarkers, compared with other biomarkers (e.g., CSF), showed higher correlation with concurrent change on general cognitive and functional indices in impaired subjects. 

**Figure 9:**
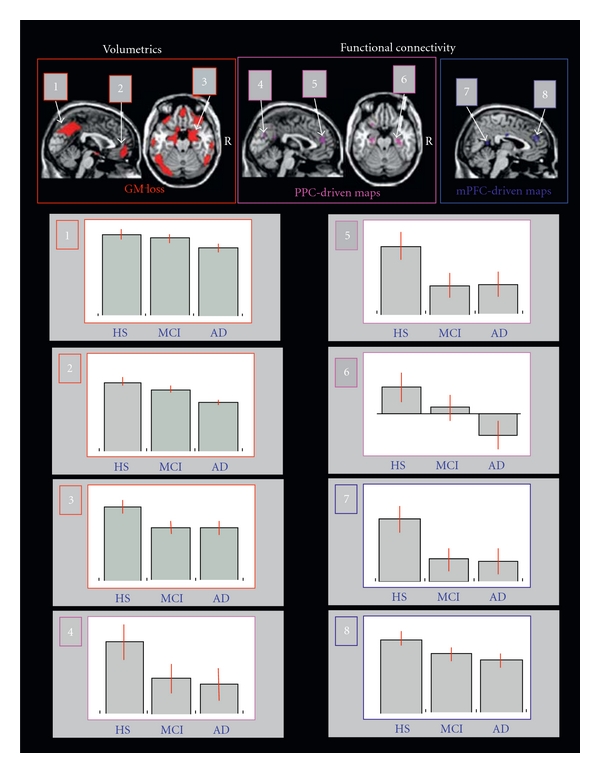
Distribution of reduced regional GMV (top panel on the left) and functional connectivity (top panel on the right) observed in patients with fully developed dementia (AD) compared with healthy subjects (HS). Changes of functional connectivity were assessed using both posterior cingulate (PCC) and medial prefrontal cortex (mPFC) driven connectivity maps (adapted with permission from [36]).

Functional abnormality has also been shown in aMCI patients. Compared with healthy controls, aMCI patients had a regional pattern of brain disconnection between the posterior cingulate cortex (PCC) and the medial prefrontal cortex and the rest of the brain. These disconnections could be observed even in the absence of GM atrophy (Figure 9) [36].

### 4.2. Conversion from aMCI to AD

Cognitively normal elderly subjects convert to AD at a rate of only 1-2% per year, whereas aMCI subjects convert to AD at a rate of 12–15% per year [54]. Studying the similarities and differences between aMCI and AD would provide valuable information of the disease mechanism and progression. Multimodal MRI offers noninvasive methods for detection and possibly prediction of the conversion from aMCI to AD [6–8]. In a 3-year followup of 118 aMCI individuals who progressed to a diagnosis of AD, Desikan et al. reported that atrophy in the medial temporal cortex (as measured by hippocampal volume, entorhinal cortex thickness, amygdala volume, temporal pole thickness, and parahippocampal gyrus thickness) can accurately and reliably predict time to disease progression [6, 7]. They demonstrated that aMCI individuals with significant atrophy of the medial temporal factor regions are three times as likely to progress to AD, compared with aMCI individuals with preserved medial temporal factor regions. Their results also demonstrated that amygdala and temporal pole may be additional important structures for predicting the conversion from aMCI to AD. Similar observations were found in a meta-analysis involving 40 studies of imaging data from 1351 patients, suggesting that atrophy in the (trans) entorhinal area and hippocampus most reliably predict the progression from aMCI to AD [55]. These data provide strong evidence that AD-related volume losses are most readily detected in the medial temporal lobe in aMCI. The reduction in medial temporal lobe volume is therefore an important indicator in predicting the transition of aMCI to AD.

MR-based ASL techniques provide a functional biomarker (perfusion) to predict the progression from MRI to AD. In a longitudinal study (2.7 ± 1.0 years), Chao et al. reported that the MCI individuals who converted to dementia displayed hypoperfusion in the right precuneus, right inferior parietal cortex, and right middle frontal cortex [56]. A similar finding was reported in the Schroeter et al. meta-analytic study (involving 1351 patients) in which hypoperfusion in the inferior parietal lobules was found to most reliably predict the progression from aMCI to AD [55]. Furthermore, baseline perfusion from the right precuneus predicted subsequent declines in clinical dementia rating sum of boxes, functional activates questionnaire and selective attention (Stroop Switching), and baseline perfusion from the right middle frontal cortex predicted subsequent episodic memory decline. These results suggest that hypoperfusion as detected by ASL MRI can predict progression from MCI to AD.

## 5. Alzheimer's Disease

**Figure 10:**
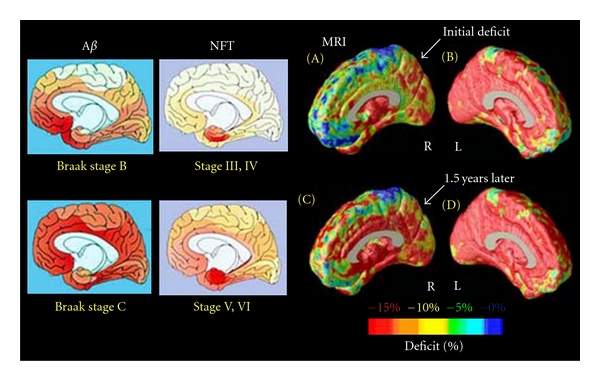
Gray matter deficits spread through the limbic system in moderate AD. Average maps of gray matter density in patients and controls were subtracted at their first scan (A and B) and at their followup scan 1.5 years later (C and D). Colors show the average percentage loss of gray matter relative to the control average. Profound loss engulfs the left medial wall (*>*15%; B and D). On the right, however, the deficits in temporoparietal and entorhinal territory (A) spread forward into the cingulate gyrus 1.5 years later (C). Limbic and frontal zones clearly show different degrees of impairment (C). MRI-based changes, in living patients, agree strongly with the spatial progression of *β*-amyloid (A*β*) and NFT pathology observed after mortem (Braak Stages B, C, and III to VI), (adapted with permission from [37, 38]).

AD is histopathologically characterized by the formation of *β*-amyloid (A*β*) plaques and neurofibrillary tangles (NFT). Progressions of the A*β* plaques and NFT pathology of AD correlate closely with loss of neurons and synapses [57]. These losses further result in gross atrophy, including cortical gray matter loss, reduced subcortical gray, and white matter volumes, as well as expanding ventricular and sulcal cerebrospinal fluid (CSF) spaces [37, 38] (Figure 10; similar regions of A*β*/NTF deposition and brain atrophy in AD). In AD, this brain atrophy is localized to the medial temporal limbic cortex during its earliest states. At later stages of disease, it progresses to paralimbic cortical regions and the neocortex [57]. The temporal limbic cortex has essential roles in episodic memory. Since memory impairment is the earliest symptom of AD, the temporal limbic cortex (including entorhinal cortex and hippocampus) has been an attractive target for structural neuroimaging studies [58–62]. 

Using brain volumetric measurements, patients with mild AD showed significantly smaller brain regions of hippocampus (25%) and entorhinal cortex (37%) than healthy elderly controls [58–62]. In a number of longitudinal studies, significantly higher rates of brain atrophy were observed in AD [63–66]. The global atrophy rate in normal aging typically increases from 0.3% to 0.5% per year at age 70–80 but increases from 2% to 3% per year in AD [67–69]. Similar regional observations have also been found in hippocampus (controls, 1.0% to 1.7% per year; AD, 3.0% to 5.9% per year) and in entorhinal cortex (controls, 1.4% to 2.9% per year; AD, 7.15 to 8.4% per year) [2, 70, 71].

MR-based volume measures, particularly for the hippocampus, have been shown to be a strong structural biomarker for AD, as follows [72–74]. First, it has been demonstrated that a significant correlation exists between MRI and histological-based hippocampal volumes (*r* = 0.97, *P* < 0.001); the difference in the hippocampal volumes between normal and AD groups was 42% for the MRI data, and 40% for the histology data after adjusting for tissue shrinkage during specimen processing. Moreover, both the histological and MRI hippocampal volume measurements were significantly associated with the number of hippocampal neurons (*r* = 0.91, *P* < 0.001 and *r* = 0.90, *P* < 0.01). Second, when compared with a temporal lobe neocortical reference volume, the hippocampal volume showed an anatomically unique correlation to memory performance such as delayed verbal recall [72–74]. 

**Figure 11:**
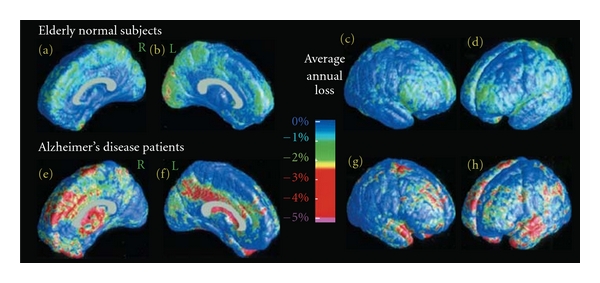
Average gray matter loss rates in healthy aging and AD. The maps show the average local rates of loss for gray matter, in groups of controls (top, (a)–(d)) and patients with AD (bottom, (e)–(h)). Loss rates are <1% per year in controls. They are significantly higher in AD and strongest in frontal and temporal regions (g, h) at this stage of AD. (adapted with permission from [39]).

With GMT measurements, mild-to-moderate AD subjects have cortices that are an average of 18% thinner relative to healthy controls (AD = 3.1 ± 0.28 mm, controls = 3.74 ± 0.32 mm) [75]. Significant GMT declines in AD were found in temporal, orbitofrontal, and parietal regions. The most pronounced changes occur in the allocortical region of the medial temporal lobes, which outlines the parahippocampal gyrus representing a loss of *>*1.25 millimeters of cortical thickness [75, 76]. In a followup study 1.5 years later, patients with AD lost significant GM (*P* < 0.05 for overall annual loss of gray matter) (Figures 11(e)–11(h); [39]) at a significantly higher rate than controls (*P* < 0.042), with a total gray matter loss rate of 5.03 ± 2.28% per year (left hemisphere 5.43 ± 3.29% per year; right hemisphere 4.64 ± 3.31% per year, whereas few regions in healthy controls exceeded a 1% annual gray matter loss). Regions with a prominent 4-5% annual loss included the right cingulate, temporal, and frontal cortices bilaterally (Figure 11, bottom row).

**Figure 12:**
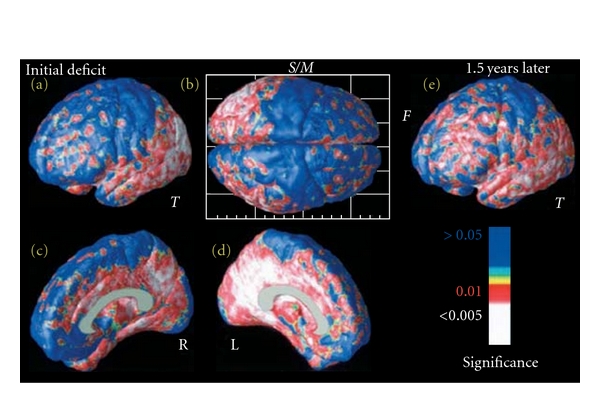
Mapping links between cognitive performance and changing brain structure. These maps show the significance of the linkage between gray matter reductions and cognition, as measured by MMSE score. Variations in temporal, parietal, and ultimately frontal (e) tissue are linked with cognitive status. Less gray matter is strongly correlated with worse cognitive performance, in all regions with prominent deficits. Linkages are detected most strongly in the left hemisphere medial temporoparietal zones (d). As expected, no linkages are found with sensorimotor gray matter variation (b), which was not in significant deficit in late AD (adapted with permission from [39]).

GMT changes are strong structural biomarkers for AD. Highly significant linkage was found relating greater GMT deficits to lower cognitive scores on the MMSE (Figure 12; [39]). These correlations were observed in all brain regions, including the temporal, parietal, and limbic cortices. Correlations were also found between frontal gray matter reduction and lower MMSE scores, but only at the later time point, when frontal gray matter was in significant deficit (Figure 12(e)). No correlations were found between gray matter differences in sensory and motor cortices and cognitive performance (Figure 12(b), blue, *S*/*M*). These results support the theory that the relationship between brain structure and cognition is regionally specific in AD, at least initially (Figure 12(b)). Correlations were observed to be strongest in regions with greatest average loss, such as the left cingulate and left temporal and parietal cortices (Figure 12(d)).

**Figure 13:**
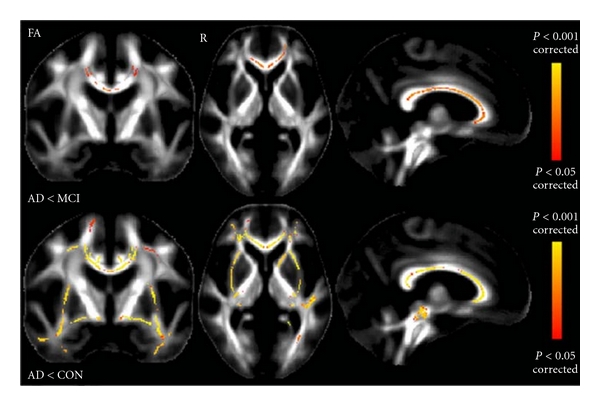
First row: significant FA results showing the contrast MCI > AD; second row: significant FA results showing CON (controls) > AD (adapted with permission from [34]).

**Figure 14:**
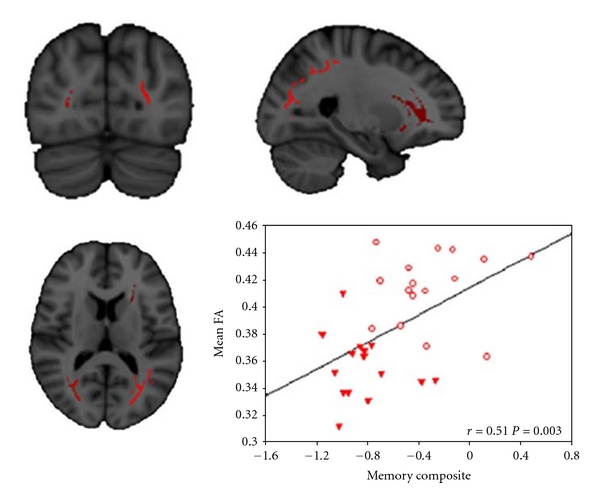
Significant correlations between regional alterations of FA in patients and the composite memory scores (adapted with permission from [40]).

WM degeneration has also been considered an important indicator of AD. In a comparison of healthy young versus older adults, WM has been found to decline in volume with increasing age but is further reduced in AD, with parahippocampal, entorhinal, inferior parietal, and rostral middle frontal areas showing the strongest AD-associated reductions in WM [77]. AD patients, similar to aMCI patients (but more severe), have shown significant increase in diffusion atrophy in the region of crossing fibers in the centrum semiovale (Figure 6; [34]), They have further shown regionally specific shape abnormalities and reduction in fractional anisotropy (FA) in the corpus callosum, anterior commissure, uncinate fasciculus, cingulum tract, and sagittal stratum tract (these have not observed in aMCI patients in comparison to NC; Figure 13; [34, 78, 79]). These results suggest that disruption in the white matter tracts near the temporal lobe may represent the secondary consequence of the medial temporal lobe pathology in AD. This is consistent with the observation that FA values are significantly related to memory performance among AD patients (Figure 14; [40]). 

Structural imaging is able to detect AD only at a stage in which the disease has progressed so far that neurons are already irreversibly lost. Ideally, AD should be diagnosed at an earlier stage in which neurons are impaired by the disease process, but not yet fully damaged, and thus can be potentially salvaged [2, 13]. In contrast, alterations of neuronal activity, metabolism, and hemodynamics are accompanied by the impairment of neurons and usually precede neuronal death prior to any cognitive deficits. Functional MRI has shown great promise in the detection of AD at this very early stage of disease, as well as during disease progression. 

**Figure 15:**
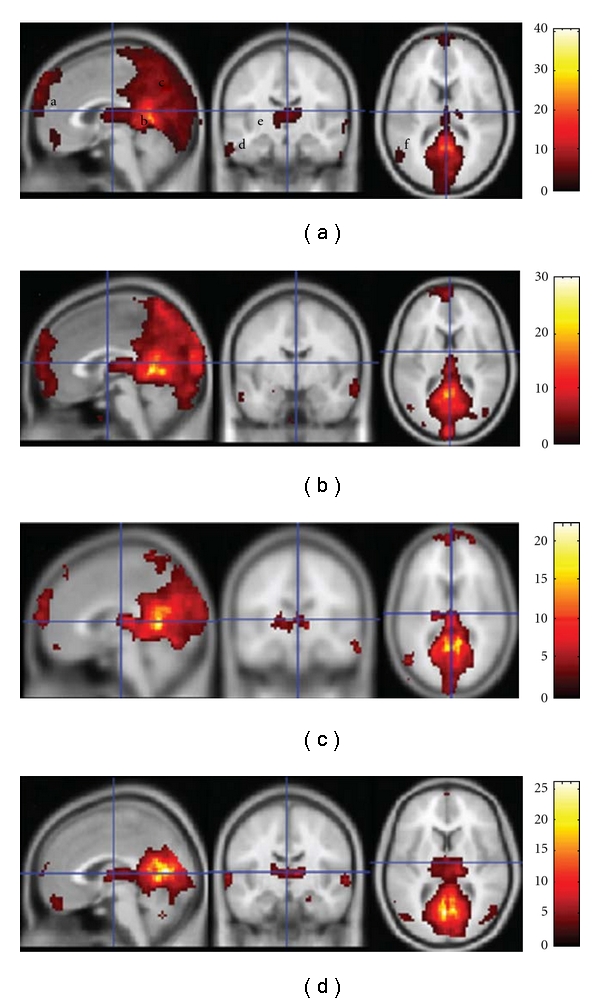
(a–d) Left to right: sagittal, coronal, and axial views of T1-weighted MNI canonical brain templates show intragroup maps of the default mode network (DMN) based on the seed of the PCC in, (a), control group, (b), mild AD group, (c), moderate AD group, and (d), severe AD group. The regions involved in the DMN are labeled as follows in (a): a = ventral MPFC, b = PCC, c = precuneus and/or cuneus, d = ITC, e = thalamus, and f = inferior parietal cortex. Color scale = *t* values (adapted with permission from [41]).

Disrupted functional connectivity has been observed in patients with AD in the default mode network, which is associated with autobiographical memory retrieval [80, 81]. Similar to aMCI, functional connectivity abnormalities were observed in the PCC and a set of default mode regions, including the medial prefrontal cortex, hippocampus, inferior temporal cortex, bilateral visual cortices, and the precuneus in AD patients [82]. This disruption of connectivity was observed to intensify during aMCI and AD disease progression (Figure 15; [41]). With concurrent fcMRI and structural MRI measurements, the PCC showed reduced connectivity in patients progressing into AD even in the absence of GM atrophy (Figure 9; [36]). This indicates that functional connectivity abnormalities precede GM atrophy in the PCC and supports the hypothesis that GM atrophy in specific regions of AD brains likely reflects a long-term effect of brain disconnection.

In AD patients, significant declines of CBF have been found in frontal, parietal, and temporal regions (*P* < 0.001), with more marked reductions in those patients with severe dementia. Covariance analysis revealed that aging and disease severity have a pronounced effect on CBF, especially that of the left parietal region. Significant decreases of CBF have been detected with ASL-MRI in temporal, parietal, and frontal cortex and the posterior cingulate in patients with AD, compared to the healthy elderly controls. The observations have been consistent with those observed using SPECT. Using dynamic contrast method, CBF has been found significantly reduced in insular cortex [83, 84]. Because the insula is an important brain structure for the autonomic control of blood pressure and heart rate, the observations suggested that AD pathology has effect on ventral autonomic cardioregulatory dysfunction.

In AD patients, significant N-acetyl aspartate (NAA) reductions have been found in various brain areas, including PCC, hippocampus, and GM of the temporal, parietal, and sometimes the frontal as well as occipital cortices [23, 85]. Decrease in NAA reflects a combination of losses of neuronal cells/dendritic structures, reduced myelination, and decreased neuronal metabolism. As a result, the degree of cognitive impairment has been well correlated with the degree of NAA decrease; poor performance on memory tests correlated with lower gray matter NAA level [86]. In contrast to NAA, myoinositol (MI), a glial biomarker, has been found to be dramatically increased in AD. Elevated MI is most likely due to the increase of gliosis in AD. Taken together, NAA and MI are important and useful metabolic biomarkers to distinguish AD from normal aging.

## 6. Future Directions

Mitochondrial dysfunction is a well-known biomarker of AD. Mitochondria are the predominate source (>98%) of energy production in mammals, yielding ATP through oxidative phosphorylation of glucose. In the brain, oxidative metabolism (O_2_ consumption) is the predominant source of energy (ATP generation), supporting baseline demands and maintaining viability, as well as responding rapidly and in a highly regional manner to changes in neuronal activity induced by task performance. Failure to maintain adequate levels of tissue oxygenation rapidly results in tissue death as observed of brain atrophy in AD patients. 

To identify the integrity of the mitochondrial function, cerebral metabolic rate of glucose (CMR_Glc_) and oxygen (CMRO_2_) are the most-well known indicators [87]. CMR_Glc_ measurements in AD research have been well established with positron emission tomography (PET) methods. For instance, significant decreases of glucose metabolism have been found in young APOE *ε*4 carriers (30 years old) in brain areas associated with AD pathology [88]. In contrast, CMRO_2_ measurements are not feasible using PET methods, especially for AD patients, due to the difficulties of obtaining arterial blood samples. In addition, the radioactive nature of PET allows less repetitive scans, which limits the monitoring of the disease progress. Therefore, considerable efforts have been made to develop MRI-based, noninvasive, CMRO_2_ measurements. Baseline CMRO_2_ and task-induced changes in CMRO_2_ determinations have been proposed by several methods, including T_2_-relaxation-under-spin-tagging (TRUST) and quantitative BOLD (qBOLD) techniques [89–93]. These MR-based metabolic imaging methods, in addition to MRS, are expected to be very useful as diagnostic and prognostic biomarkers in AD. However, future studies allowing for rigorous assessment of test-retest reliability and power calculation compared to the established imaging techniques are necessary before these CMRO_2_ methods can be accepted as other complementary and/or established functional neuroimaging biomarkers for AD.

The development and validation of structural and functional biomarkers will enable MRI to be utilized as a powerful tool for evaluation of therapeutic efficacy in AD in large-scale clinical trials. For example, Jack et al. estimated that in each arm of a therapeutic trail with conventional volumetric measures for hippocampal volume, only 21 subjects would be required to detect 50% reduction in the rate of decline. This compares 241 subjects if MMSE scores were used; 320 subjects if ADAS Cog scores were used [54]. Combining with other biomarkers (e.g., CMR_Glc_ by PET and A*β*/NFT CSF), surrogate markers for AD progress can be identified and used for clinical/cognitive tests in clinical trials. Nonetheless, these surrogate markers must be validated to be reproducible in the treatment setting, across various types of treatments, across imaging centers, and across time. A multicenter AD research project, known as the Alzheimer's Disease Neuroimaging Initiative (ANDI) (http://adni.loni.ucla.edu/), was launched in 2004 to meet this goal.

## 7. Conclusions

The incidence of Alzheimer's disease is increasing with the extended lifespan in developed countries. The development of neuroimaging biomarkers is a pressing need to detect the early risk of AD (from NC), predict and monitor disease progression (from aMCI). Multimodal MRI methods have been developed to meet this demand by providing useful and important structural and functional biomarkers in AD. The validation of the surrogate biomarkers will have profound implications in AD clinical trials, including the prevention and deceleration of AD onset, as well as the evaluation of treatment efficacy.
